# Gender-Specific Differences in Clinical Profile and Biochemical Parameters in Patients with Cushing's Disease: A Single Center Experience

**DOI:** 10.1155/2015/949620

**Published:** 2015-05-06

**Authors:** Xiaoxia Liu, Xiaoming Zhu, Meifang Zeng, Yan Zhuang, Yiting Zhou, Zhaoyun Zhang, Yehong Yang, Yongfei Wang, Hongying Ye, Yiming Li

**Affiliations:** ^1^Division of Endocrinology and Metabolism, Huashan Hospital, Shanghai Medical College, Fudan University, Shanghai 200040, China; ^2^Division of Neurosurgery, Huashan Hospital, Shanghai Medical College, Fudan University, Shanghai 200040, China

## Abstract

Cushing's disease (CD) is remarkably prevalent among females; however, more severe clinical presentation and adverse outcomes have been found in males. The purpose of this study was to investigate the overall clinical profile and biochemical parameters in patients with CD to identify the gender differences. Here we describe our series of CD patients referred to our medical center during 2012-2013. Among 73 cases, females presented a marked preponderance compared to males. Males had significantly higher ACTH, BMI, HbA1c, systolic blood pressure, and hemoglobin than females. For the first time, the incidence of fatty liver and hepatic function was also shown to be elevated in males. Multiple linear regression analysis was performed to further investigate the correlation of risk factors with hypokalemia, HbA1c, and systolic blood pressure. Gender and serum cortisol were associated with hypokalemia. Age, gender, and serum cortisol were significantly associated with HbA1c. Additionally, only gender was significantly associated with systolic blood pressure. Regarding clinical presentation, purple striae seemed to occur more frequently in males than in females. Thus, more severe clinical presentation, biochemical parameters, and complications were found in males than in females. Clinical professionals should pay more attention to the diagnosis and management of males with CD.

## 1. Introduction

Cushing's disease (CD) is caused by a corticotroph pituitary adenoma that secretes adrenocorticotroph hormone (ACTH), resulting in chronic overproduction of cortisol by the adrenal glands. This chronic state of hypercortisolism is associated with many metabolic disorders such as central obesity, diabetes mellitus, hypertension, and dyslipidemia.

It is well accepted that the incidence of CD is higher among women than among men, with new cases diagnosed at the ratio of 3–8 : 1 [1–4]. However, women are 0.3 times less likely to have adverse outcomes in comparison to men [5]. Studies of gender differences of CD were rare until Giraldi and his colleges first focused on this area in 2003 [6]. Their research demonstrated that males with CD had a more severe clinical presentation than women, including higher ACTH, urinary free cortisol levels, and prevalence of osteoporosis, muscle wasting, purple striae, and nephrolithiasis [6]. Moreover, male patients seem to have a poorer prognosis after surgery [6, 7]. Recently, a study of gender differences in the diagnosis and complications of CD moved research on this topic forward [8]. Apart from having similar findings as Giraldi, Zilio et al. [8] found that males with CD presented a lower ACTH response to desmopressin acetate (DDAVP) stimulation and less acuity of pituitary magnetic resonance imaging (MRI) than females. Furthermore, male patients presented with more severe complications than women, including hypokalaemia, hypercoagulable state and osteoporosis at the lumbar spine, vertebral fractures, and dyslipidemia. Other investigators also found differences in hematological parameters between males and females with CD [9].

The purpose of the present study was to identify the gender differences associated with clinical presentation, biochemical parameters, and complications of CD in patients from a single center. Results of this study may assist clinical physicians with the difficulties in diagnosis and management of this disorder.

## 2. Subjects and Methods

This retrospective study included 73 patients referred to the Endocrine Department of Huashan Hospital for evaluation and diagnosis of CD from January 2012 to December 2013. Sixty females and thirteen males were enrolled. All subjects had detailed clinical evaluation by the same group of endocrinology specialists to avoid subjective influences.

### 2.1. Diagnostic Methods

The 24-hour urinary free cortisol (UFC) excretion, the overnight 1 mg dexamethasone suppression test (DST), and cortisol secretion circadian rhythm were used as first-line screening tests to identify patients with Cushing's syndrome (CS) after exclusion of exogenous glucocorticoids exposure. The diagnostic criteria included (1) elevated levels of 24-hour UFC (average of at least 2 samples); (2) failure of plasma cortisol decrease <5 *μ*g/dL after overnight 1 mg DST; and (3) impaired serum cortisol secretion circadian rhythm (samples collected at 0000, 0800, and 1600), as previously described [10–12]. Plasma and urinary cortisol levels were performed by radioimmunoassay (RIA) (Roche Diagnostics, Basel, Switzerland). ACTH-dependent CS was diagnosed based on an unsuppressed ACTH level [11]. Plasma ACTH was measured in an automated chemiluminescence immunoassay (Siemens Healthcare Diagnostics, Los Angeles, CA, USA). Inferior petrosal sinus sampling (IPSS) was performed in patients with negative MRI images or pituitary tumor lesions less than 6 mm (39/73). CD was clinically diagnosed by lesion >6 mm in pituitary MRI scan [12] and positive IPSS results. CD was then confirmed histologically after transsphenoidal surgery (positive immunochemistry staining with ACTH).

### 2.2. Clinical and Biochemical Methods

Body mass index (BMI) and blood pressure were measured under the same conditions at diagnosis. Hemoglobin, plasma potassium (plasma K), hepatic function, creatinine (Cr), hemoglobin A1c (HbA1c), lipid profile, and testosterone were evaluated by standard methods. Ultrasound was used to assess fatty liver. Fatty liver was defined by the presence of at least two of three abnormal findings on abdominal ultrasonography including diffusely increased liver near-field ultrasound echo, liver echo greater than kidney; vascular blurring and the gradual attenuation of far-field ultrasound echo [13].

### 2.3. Statistical Analysis

Continuous data are shown as median and range (minimum and maximum values) and compared between groups using the nonparametric Mann-Whitney *U* test. Categorical variables were evaluated with Chi-squared test, as appropriate. Multiple linear regression analysis was used to assess associations between variables. Data were analyzed using the statistical software SPSS 10.0 (SPSS, Chicago, IL, USA). A two-tailed *P* value less than 0.05 was established as statistical significance.

## 3. Results

**Figure 1:**
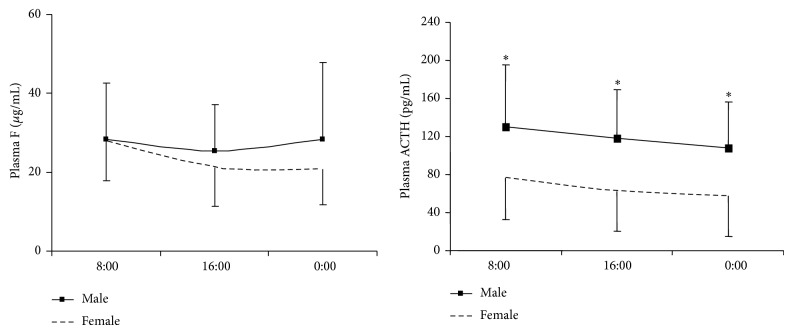
Diurnal plasma cortisol and ACTH curves in male and female patients with CD (^∗^ *P* < 0.05, Mann-Whitney *U* test).

Gender-related patients' characteristics are presented in Table 1. Overall, females (60/73) were more likely to have CD than males (13/73), with a ratio of 4.6 : 1. Most patients were diagnosed at the age of 30. No significant differences in age were found between male and female patients. The 24-hour UFC values ranged broadly across subjects with no significant difference between genders. Male patients had significantly higher BMI, HbA1c, systolic blood pressure, ALT, AST, GGT, and hemoglobin compared to female patients. Comparison of values for diurnal plasma cortisol and ACTH curves between male and female CD patients revealed that ACTH values were significantly higher in males but no differences were found in plasma cortisol between males and females (Figure 1).

**Figure 2:**
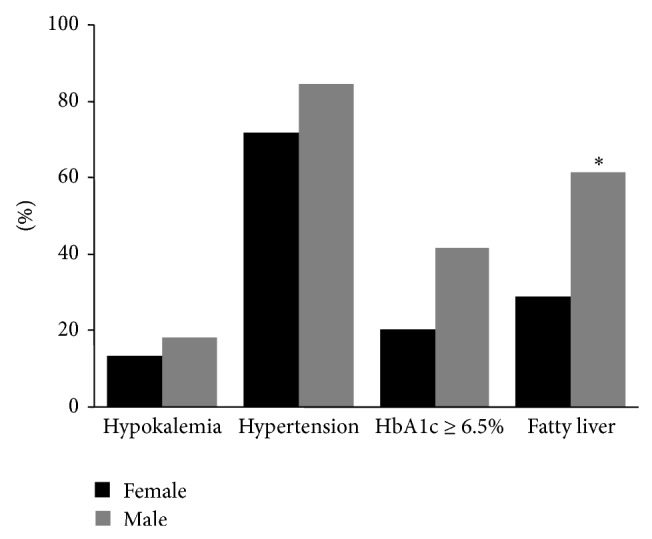
The incidence of hypokalemia, hypertension, HbA1c above 6.5%, and fatty liver in males and females with CD (^∗^ *P* < 0.05, Chi-squared tests).

Plasma K <3.4 mmol/L was considered to be hypokalemia. The incidence of hypokalemia was similar between genders (13.3% in females versus 18.2% in males, NS). Arterial hypertension was defined as blood pressure >140/90 mmHg or patients were already taking antihypertensive drugs. No significant differences were found in the prevalence of hypertension between genders (71.6% in females versus 84.6% in males, NS). Similarly, no differences were found in the frequency of HbA1c above 6.5% between genders (20% in females versus 41.6% in males, NS). Fatty liver assessed by ultrasound was found to be more prevalent in males than in female patients (28.6% in females versus 61.5% in males, *P* < 0.05) (Figure 2).

When age, gender, BMI, and plasma F values were included in the multiple linear regression model (Table 2), gender (*β* = −0.522, *P* = 0.025) and plasma F (*β* = −0.016; *P* = 0.037) were found to be associated with hypokalemia. Age (*β* = 0.086; *P* = 0.000), gender (*β* = 1.673; *P* = 0.022), and plasma F (*β* = 0.072; *P* = 0.003) were significantly associated with HbA1c. However, in multiple linear regression analysis, there did not appear to be a relationship between plasma F and systolic blood pressure. Only gender (*β* = 11.567; *P* = 0.033) was significantly associated with systolic blood pressure.

Regarding clinical presentation, the prevalence of purple striae seemed to occur more frequently in males than in females (Table 3). Meanwhile, no gender differences were found in the occurrence of central obesity, acne, headache, eye disease, weight gain, and lower extremity edema.

## 4. Discussion

In this study, to establish a better understanding of CD, we retrospectively analyzed the differences in clinical presentation, biochemical parameters, and complications between male and female CD patients. The subject population of our investigation was diagnosed and treated in only one medical center within a two-year period. Although the number of patients was smaller compared to other studies [6], one strength of our study was that we guaranteed that patients were evaluated by similar diagnostic methods such as physical examination and biochemical assessments. We investigated patients' precise clinical profile and biological parameters, presenting not only the differences but also an assessment of related risk factors.

Results of the present study confirmed that, just like many other endocrine disorders, CD was more prevalent in females. Nevertheless, previous studies have shown the opposite results among prepubertal patients [14, 15]. We did not analyze the patients by different ages since we only had data for 8 patients under 20 years, including 5 females (8.3%, range: 16 to 19 years) and 3 males (23%, range: 14 to 16 years). In spite of this, no differences were found between males and females on the average onset age, which was same as that shown by Zilio et al. [8]. However, other studies presented the opposite results for gender-related age differences [6, 7].

CD is associated with high levels of cortisol and ACTH. In the present study, the plasma and urine cortisol levels were slightly higher in male patients, but there were no statistically different results between genders. The limited number of male patients may explain the wide range of 24-hour UFC values found in this study. It would be worth assessing cortisol values in larger populations to confirm these findings in the future. In contrast to wide-ranging UFC values, ACTH levels were extremely and consistently higher in males at three different time points of a day, which agrees with results of previous studies. It has been demonstrated that pituitary adenomas in males have a greater secretory capacity, but it is still unclear what the risk factors are for CD. Also, the greater secretory capacity cannot be explained simply by the size of the adenoma. Several factors were observed that could affect the MRI diagnosis, so observations in this area are in conflict with results of other studies [6–8]. Therefore, the mechanism behind higher ACTH values in males with CD requires additional investigation.

The elevated cortisol levels were combined with several severe clinical presentations. Results of the present study highlight the differences in the clinical profiles between male and female patients at diagnosis. Our observations have confirmed that the purple striae represent a marked male preponderance of this characteristic, which had been previously reported [6]. This finding indicated that hypercortisolism represents the greater inhibitory activity of fibroblasts occurring in men compared to that in women.

As is already well known, the glucocorticoids are important modulators of red blood cell development [16]. Patients with CD are often described as being polycythemic and plethoric [17]. Results of the present study have indicated that the hemoglobin concentration is greater in men than in women. This is contrary to previous reports showing that men with CD have low-to-normal hemoglobin concentration [9]. This difference greatly depends on the testosterone levels. Several studies have demonstrated that testosterone has effects on erythropoiesis [18, 19]. In the present study, testosterone concentrations were within a normal range, whereas hypogonadism was diagnosed in all but three male patients in the study of Ambrogio et al. [9].

Similar to ectopic ACTH secretion, the prevalence of hypokalemia at diagnosis correlated with cortisol levels [20]. This may possibly be explained by that fact that excess cortisol can saturate the cortisol inactivating enzyme (11*β*-hydroxysteroid dehydrogenase type 2, 11*β*-HSD2) to bind with mineralocorticoid receptors that exhibit mineralocorticoid activity [20, 21]. When we put cortisol values and gender together, the hypokalemia represents a significant gender-related difference, despite the fact that there was an apparently similar degree of hypercortisolism and incidence of hypokalemia between genders. These findings have since been verified by other investigators [7, 8]. In addition, many retrospective research data suggest that a high proportion of adult patients with CS suffer from hypertension [22, 23]. Interestingly, the specific feature of CS-induced hypertension is the lack of a significant difference between genders or among the different etiologies of CS [6, 8, 22, 23]. Moreover, the blood pressure values were not found to be associated with circulating cortisol levels and BMI [20, 22, 24–27]. Some researchers have deduced that males have higher blood pressure based on the numbers of antihypertensive drugs used by male patients [8]. Data of the present study has confirmed previous reports on the incidence and risk factors of hypertension in CD. In addition, and for the first time, this study has shown a difference in the prevalence of overall systolic blood pressure between genders. The duration of hypercortisolism seemed to correlate with the development of hypertension [22]. Furthermore, the elevated levels of ACTH in males appear to be much more important to their overall condition. ACTH not only can result in aldosterone release [28], but also may decrease 11*β*-HSD2 activity, which may amplify the actions of cortisol on the mineralocorticoid receptor [29]. Thus, mineralocorticoid action would appear to play a role in this study, although we failed to analyze the aldosterone levels due to the missing data of many patients. As already known, hypokalemia and arterial hypertension are strongly associated with overall CD mortality. Therefore, our findings may partly explain why men had poorer outcomes and prognosis than women.

Glucose metabolism disorders are a frequent complication of CD [30, 31]. Another finding of the present study was that the average levels of HbA1c clearly indicated gender dependence. Men with CD presented elevated HbA1c levels and women, on the contrary, presented normal levels. Further analysis of HbA1c above 6.5%, which is considered to be diagnostic criteria for diabetes [32], showed a rising tendency in males, although there were no statistically significant differences between males and females. Considering the aspect of the link between CD and glucose metabolism, unlike other investigators, our study focused on HbA1c, which provides an average of plasma glucose concentration over prolonged periods of time. Therefore, gender-related glucose metabolism disorders of CD were much more stable and convincing. This finding indicated that males had a somewhat higher risk of impaired glucose metabolism than females. Apart from gender, age and cortisol concentration also appeared to correlate strictly with HbA1c levels. Giordano et al. [33] carefully conducted phenotypic evaluation of glucose tolerance defects in patients with CS, which confirmed our findings. They reported that age, genetic predisposition, and lifestyle, in combination with the duration and degree of hypercortisolism, strongly contribute to the impairment of glucose tolerance in patients with a natural history of CS [33]. Regarding lipid metabolism, which is another common metabolic disorder of CD, we did not find any differences in lipid-related molecules in plasma between genders. The utilization of antihyperlipidemia drugs might affect this analysis. Another limitation of the present study is that visceral obesity was not included in the evaluation due to missing data from many patients about waist and hip circumference. In spite of this, the higher prevalence of BMI and fatty liver in males with CD indicated that males were at higher risk of developing lipid metabolic disorders. Moreover, male sex has been found to be an independent risk factor for triglycerides and HDL levels in CD patients [8].

A final gender-related issue of hepatic function is worth mentioning. The glucocorticoids, as well known, have strong effects on the liver that affects the secretion of several hormones. Our findings documented elevated levels of biologic markers for liver function in men, which has not been reported previously. The higher frequency of BMI found in males could be an important factor. Also, the presence of fatty liver might partially explain this result. The diagnosis of fatty liver using ultrasound revealed a higher prevalence in males than in females. However, previous reports showed that only 20% of patients with CS had hepatic steatosis using computed tomography (CT) and there were no gender differences [34]. In this regard, the different types of assessments used for diagnosis and differences in patients' lifestyles should be considered. Another interesting finding was serum creatinine (Cr), which was used to assess overall renal function. In spite of the gender-related significant differences, the levels of Cr were at normal range in both groups. Another study reported that CS might cause a decreased GFR in combination with a higher cardiovascular risk [35]. Therefore, putting the above findings together suggests that drug treatment of CD should be more carefully considered in males.

In conclusion, the results of this current investigation demonstrated that males with CD were fewer than females but had more severe clinical presentation, biochemical parameters, and complications, including ACTH, systolic blood pressure, HbA1c, BMI, hepatic function, and fatty liver. Results of this study may help to draw physicians' attention to the diagnosis and management of CD in males.

## Figures and Tables

**Table 1:** Clinical features of CD by gender.

	Females (*n* = 60)	Males (*n* = 13)	*P* value
Age (years)	33.5 (16–62)	30.0 (14–64)	0.762
BMI (kg/m^2^)	24.0 (17.1–34.4)	27.2 (20.8–35.8)	0.076
Plasma K^+^ (mmol/L)	4.1 (2.9–5.2)	3.9 (3.0–4.7)	0.310
Systolic blood pressure (mmHg)	135.5 (100.0–170.0)	152.0 (120.0–164.0)	0.022
Diastolic blood pressure (mmHg)	92.0 (62.0–129.0)	99.5 (80.0–110.0)	0.086
HbA1c (%)	5.6 (4.7–11.0)	5.8 (4.6–10.0)	0.118
24 h UFC (g/24 h)	442.0 (241.2–844.4)	671.9 (161.4–1618.2)	0.497
T (nmol/L)	1.8 (0.09–5.13)	4.4 (1.65–12.03)	0.001
Hemoglobin (g/L)	141.0 (98.0–164.0)	156.0 (138.0–169.0)	0.001
ALT (U/L)	26.5 (10.0–103.0)	50.5 (20–405.0)	0.002
AST (U/L)	16.5 (10.0–34.0)	25.5 (13.0–58.0)	0.002
GGT (U/L)	20.5 (10–112)	60.0 (13–295)	0.001
Cr (*μ*mol/L)	54.5 (38–85)	67.0 (56–85)	0.000
CHO (mmol/L)	5.3 (3.1–9.0)	4.9 (3.51–7.13)	0.952
TG (mmol/L)	1.4 (0.54–4.59)	1.7 (1.07–3.83)	0.095
HDL (mmol/L)	1.4 (0.83–2.72)	1.3 (0.96–1.84)	0.667
LDL (mmol/L)	3.3 (1.62–6.02)	3.4 (1.6–4.8)	0.786

Data are expressed as median and range. T, testosterone; ALT, alanine transaminase; AST, aspartate transaminase; GGT, gamma-glutamyl transaminase; CHO, cholesterol; TG, triglyceride; HDL, high-density lipoprotein; LDL, low-density lipoprotein; Mann-Whitney *U* test.

**Table 2:** Linear regression analysis for hypokalemia, HbA1c, and systolic blood pressure.

Model	Variables	*β*	S.E.	*P* value	95% CI
Hypokalemia	Age	−0.010	0.007	0.171	−0.024~−0.004
	Sex	−0.522	0.227	0.025	−0.975~−0.068
	BMI	0.010	0.019	0.599	−0.028~−0.048
	Plasma F	−0.016	0.008	0.037	−0.031~−0.001

HbA1c	Age	0.086	0.022	0.000	0.042~0.129
	Sex	1.673	0.711	0.022	0.252~3.094
	BMI	0.013	0.060	0.831	−0.107~0.132
	Plasma F	0.072	0.024	0.003	0.025~0.119

SBP	Age	0.298	0.164	0.074	−0.029~0.626
	Sex	11.567	5.314	0.033	0.948~22.186
	BMI	0.524	0.447	0.245	−0.369~1.417
	Plasma F	0.280	0.176	0.117	−0.072~0.631

SBP, systolic blood pressure.

**Table 3:** Differences in clinical presentation of CD by gender.

	Females	Males	*P* ^b^
Central obesity	52 (86.7%)	11 (84.6%)	0.569
Purple striae	20 (33.3%)	9 (69.2%)	0.016
Acne	19 (31.7%)	5 (38.5%)	0.432
Headache	2 (3.3%)	1 (7.7%)	0.473
Eye disease^a^	4 (6.7%)	2 (3.3%)	0.289
Weight gain	27 (45%)	7 (53.8%)	0.391
Lower extremity edema	12 (20%)	0 (0%)	0.078

^a^Including impaired vision and visual fields. ^b^ *P* for Chi-squared test.
